# Mud extrusion and ring-fault gas seepage – upward branching fluid discharge at a deep-sea mud volcano

**DOI:** 10.1038/s41598-018-24689-1

**Published:** 2018-04-19

**Authors:** M. Loher, T. Pape, Y. Marcon, M. Römer, P. Wintersteller, D. Praeg, M. Torres, H. Sahling, G. Bohrmann

**Affiliations:** 1 MARUM – Center for Marine Environmental Sciences and Department of Geosciences at University of Bremen, Klagenfurter Str, 28359 Bremen, Germany; 2 OGS (Istituto Nazionale di Oceanografia e di Geofisica Sperimentale), Borgo Grotta Gigante 42/c, Sgonico, 34010 Trieste, Italy; 3 College of Earth, Ocean, and Atmospheric Sciences, Oregon State University, 104 CEOAS Administration Building, Corvallis, OR 97331-5503 USA; 4 Present Address: Institute of Petroleum and Natural Resources, PUCRS, Av. Ipiranga, 6681, 90619-900 Porto Alegre, RS Brazil

## Abstract

Submarine mud volcanoes release sediments and gas-rich fluids at the seafloor via deeply-rooted plumbing systems that remain poorly understood. Here the functioning of Venere mud volcano, on the Calabrian accretionary prism in ~1,600 m water depth is investigated, based on multi-parameter hydroacoustic and visual seafloor data obtained using ship-borne methods, ROVs, and AUVs. Two seepage domains are recognized: mud breccia extrusion from a summit, and hydrocarbon venting from peripheral sites, hosting chemosynthetic ecosystems and authigenic carbonates indicative of long-term seepage. Pore fluids in freshly extruded mud breccia (up to 13 °C warmer than background sediments) contained methane concentrations exceeding saturation by 2.7 times and chloride concentrations up to five times lower than ambient seawater. Gas analyses indicate an underlying thermogenic hydrocarbon source with potential admixture of microbial methane during migration along ring faults to the peripheral sites. The gas and pore water analyses point to fluids sourced deep (>3 km) below Venere mud volcano. An upward-branching plumbing system is proposed to account for co-existing mud breccia extrusion and gas seepage via multiple surface vents that influence the distribution of seafloor ecosystems. This model of mud volcanism implies that methane-rich fluids may be released during prolonged phases of moderate activity.

## Introduction

Mud volcanoes (MVs) are geological structures created by the extrusion of sediments, water, and volatiles (predominantly methane) from subsurface plumbing systems that may extend to depths of kilometres^1–4^. Therefore, terrestrial MVs represent a significant natural source of atmospheric methane with emissions estimated to range from 6 to 12.6 Tg/year^5,6^. The jointly expelled solids and fluids are referred to as mud breccia and over time the extrusion of mud breccia can build morphological edifices up to hundreds of meters in height that cover areas of up to several square kilometres^7^. Observations of terrestrial MVs suggest that mud volcanism is episodic, with periods of quiescence that may represent up to 95% of the lifetime of a MV, interrupted by brief eruptions (hours to days)^3,7,8^. Quiescent MV activity is typically characterized by the expulsion of mud and fluids from small cones (<10 m high) or fluid-mud pools and gas seeps^8^. These features may be linked to extensional faults within the extrusive edifice, in some cases defining sub-circular calderas thought to result from post-eruptive subsidence^9^.

Less is known about the expressions of activity (quiescent or eruptive) of submarine MVs although estimates of their number (up to 10^5^) exceed that of onshore MVs by up to two orders of magnitude^5,10^. In the Black Sea, seepage from active MVs is associated with warm sediments ascending from depth and upward flow of aqueous fluids and free gas^11,12^. At the Håkon Mosby MV (Norway), variations in fluid flow have been found to trigger mud movements and outflows that changed the seafloor morphology and released up to 43000 m^3^ of methane^13,14^. Deep-sea MVs are often associated with the presence of shallow gas hydrates^12,15^ and are known to sustain chemosynthesis-based seafloor ecosystems referred to as cold seeps^16^. However, the current understanding of the mechanisms driving fluid flow, the fluid sources (i.e. deep vs. shallow), the discharge volumes of volatiles and the impact of mud volcanism on seafloor ecosystems is poorly constrained (e.g. Bohrmann *et al*.^12^). To move forward in the understanding of these interrelated issues requires systematic investigations of submarine MV systems to detect the sites of seepage relative to MV morphologies, quantify the fluid discharge, and characterize the composition of fluids emitted at and in the vicinity of the locations of active mud extrusion.

**Figure 1:**
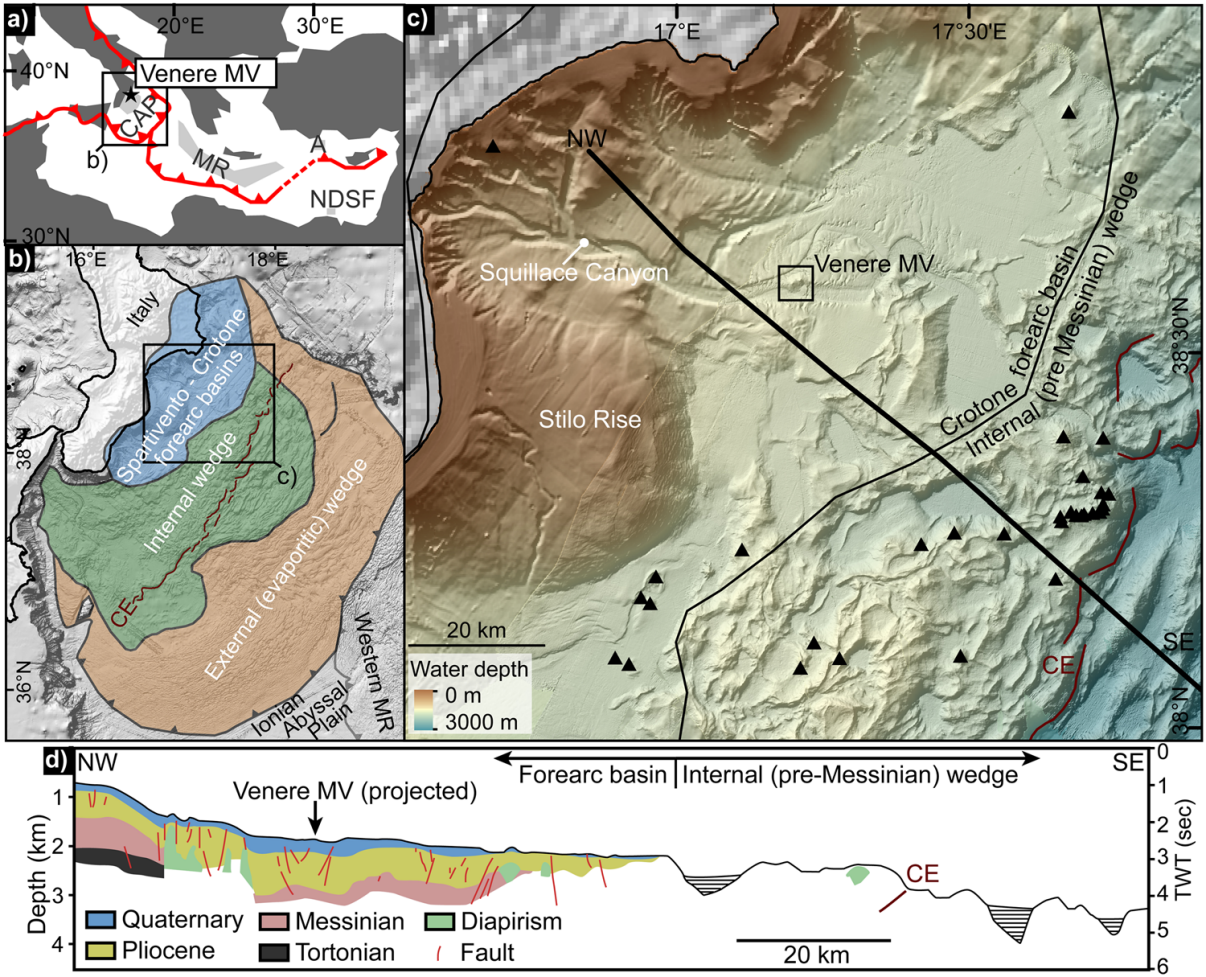
Maps and geological cross section of the study area. (**a**) Location of Venere mud volcano (MV; star) in the Calabrian accretionary prism (CAP) relative to plate boundaries (red lines) and main MV provinces (light grey shading, after Mascle *et al*.^18^) in the E Mediterranean Sea (MR = Mediterranean Ridge, A = Anaximander Mountains, NDSF = Nile Deep Sea Fan); (**b**) Main morpho-structural zones of the CAP and boundaries after Ceramicola *et al*.^25^ (see a) for extent; CE = Calabrian Escarpment); (**c**) Distribution of MVs (after Ceramicola *et al*.^25^ on the internal wedge and forearc basins (see b) for extent) including Venere MV; (**d**) NE to SW profile across the Crotone basin showing the geological context of the study area. Note that Venere MV is located 7 km from the profile; the profile was drawn according to the interpretation by Panieri *et al*.^38^ (see their Fig. 2b showing sparker seismic line J-08); depth scale on left is approximated by assuming a P-wave velocity of 1,500 m/s. Maps a) to (**e**) were generated in ESRI ArcMap 10.3.1 (www.esri.com).

MVs occur globally in a variety of plate tectonic settings, but are most common along convergent margins within accretionary prisms^3,4,17^. The eastern and central Mediterranean regions host an exceptionally high density of MVs^18^ the majority of which are located within the accretionary settings of the Africa-Eurasia subduction zone. Major MV provinces have been identified on the Calabrian accretionary prism (CAP), the Mediterranean Ridge, the Anaximander Mountains, and the Nile deep sea fan (Fig. 1a^18^). On the Mediterranean Ridge, scientific drilling of two MVs^19^ revealed mud breccia discharge processes^20^ and a fluid source depth of ~3.5–7 km^21^. In the Anaximander Mountains region, gas release at MVs supports the formation of shallow gas hydrates^22^, which have been found to be prone to decomposition during eruptive phases of mud volcanism^15^. Investigations of MVs on the Nile deep sea fan documented mud outflows and fluid seepage at submarine brine pools^23,24^. The presence of brine indicated migration from below and through fault-ruptured Messinian evaporite layers^24^. In the main sediment basins of the eastern Mediterranean, however, Messinian salt deposits are thought to act as seals to fluids ascending from depth. The MV provinces have in turn been found to predominantly occur where salt deposits are absent, thin, or undetected in seismic data^18^.

At least 54 extrusive features have been documented across the inner regions of the Calabrian accretionary prism^25^ (CAP; Fig. 1a). This includes Venere MV located in the Crotone basin (Fig. 1b) at ~1,600 m of water depth along the axis of the Squillace canyon (Fig. 1c). Systematic hydroacoustic surveys across at least 50 extrusive features previously recognized by Ceramicola *et al*.^25^ were performed in November and December 2014 (R/V METEOR cruise M112^26^). Evidence of active gas emissions into the water column (gas flares) and mud breccia outflows were found only at Venere MV^27^. A recent study on the extrusive history of Venere MV presented evidence of mud breccia discharge for at least the last 800 years at rates as high as 47000 m^3^/year, suggesting a long-term pressurized state of the MV system^27^. These findings contrast with the typical view of short-lived massive mud breccia extrusions interrupting long-term quiescent-type seepage. They require a more detailed study of the present-day activity of Venere MV, including potential fluid sources.

In this study the nature of activity at a submarine MV is investigated, based on a multi-method approach involving hydroacoustic and visual seafloor datasets acquired by AUV (autonomous underwater vehicle) and ROV (remotely operated vehicle) surveys, seafloor sediment samples, gas samples collected under *in-situ* pressure, and fluid analyses. The multi-parameter datasets are used to study processes involved in active mud volcanism. The processes that drive mud volcanism typically occur at depths not readily accessible for direct sampling, thus the results allow for inferences on crustal dewatering and hydrocarbon generation at depth within the CAP. Element and fluid cycling and contribution of elements to the hydrosphere, which may be applicable to other convergent margins are discussed. Finally, the findings of this study support a new conceptual model involving coeval mud breccia extrusion, faulting and gas seepage, in which subsurface fluid migration pathways are linked to different expressions of seafloor fluid discharge and ecosystem distribution.

## The Calabrian accretionary prism and mud volcanoes

The present-day CAP in the central Mediterranean Sea (Fig. 1a) developed from the NW subduction of the African plate (an Ionian lithospheric slab) below the Eurasian plate during the Neogene^28,29^. The incorporation of evaporites resulting from the Messinian salinity crisis (late Miocene) into the accretionary prism strongly influenced the structure of the CAP. The CAP can be divided into an external (post-Messinian) wedge that hosts evaporites and an internal (pre-Messinian) wedge hosting mainly clastic sediments affected by out-of-sequence thrusts, the most prominent of which is referred to as the Calabrian Escarpment (CE; Fig. 1b^30–32^). The internal wedge is composed of accreted Mesozoic-Cenozoic units overlain by forearc basins (Spartivento-Crotone basins^25,30–33^). Late Pleistocene uplift has resulted in exposure of the inner parts of the forearc basins in southern Calabria, where sediments date back to the middle Miocene (late Serravallian) and contain Messinian deposits including <200 m-thick evaporites within the onshore Crotone forearc basin^34,35^ and in nearshore wells^36^. Seismic reflection profiles of the offshore forearc basins indicate up to 2 km of sediment, overlying thrust structures (Fig. 1d^31,32,37,38^). These sequences are inferred to also date back to the Tortonian and to contain Messinian deposits^30–32,36^. However, the sedimentological composition of these deposits – i.e. whether they contain evaporites – remains unknown.

The majority of the 54 MVs so far identified in the CAP are located across the internal (pre-Messinian) accretionary prism, within the forearc basins and among the thrust-fold belt to seaward^25^. MVs in both tectonic settings are argued to have been erupting since ca. 3.5 Ma (early Pliocene), based on seismic profiles across two structures. These two MVs are associated with buried extrusive edifices over 1 km thick that interfinger with the upper part of the Plio-Quaternary succession^2^. The geometries of these and other MVs have been argued to reflect the interaction of fluids rising from within the prism with near-surface structures, including thrusts beneath the forearc basins^2,25^. Mud breccia from several MVs contained sediment derived from strata ranging in age from Recent to as old as Late Cretaceous, consistent with sources at depth within the accretionary prism^2,38,39^. Most MVs are associated with seafloor backscatter signatures interpreted to indicate mud breccia outflows over the last glacial to interglacial cycle^25^. Evidence of presently ongoing fluid release on the CAP is limited, however, to reports of sediment cores releasing gas and findings of small tubeworms in a single core^40^. Previous studies of MVs on the CAP lack a quantification of the gas or characterization of the involved fluids. Cold seep ecosystems represent important ecological hotspots in the Mediterranean Sea but they are still poorly known on the CAP.

## Extrusive morphology of Venere MV – ring-faults and mud outflows

**Figure 2:**
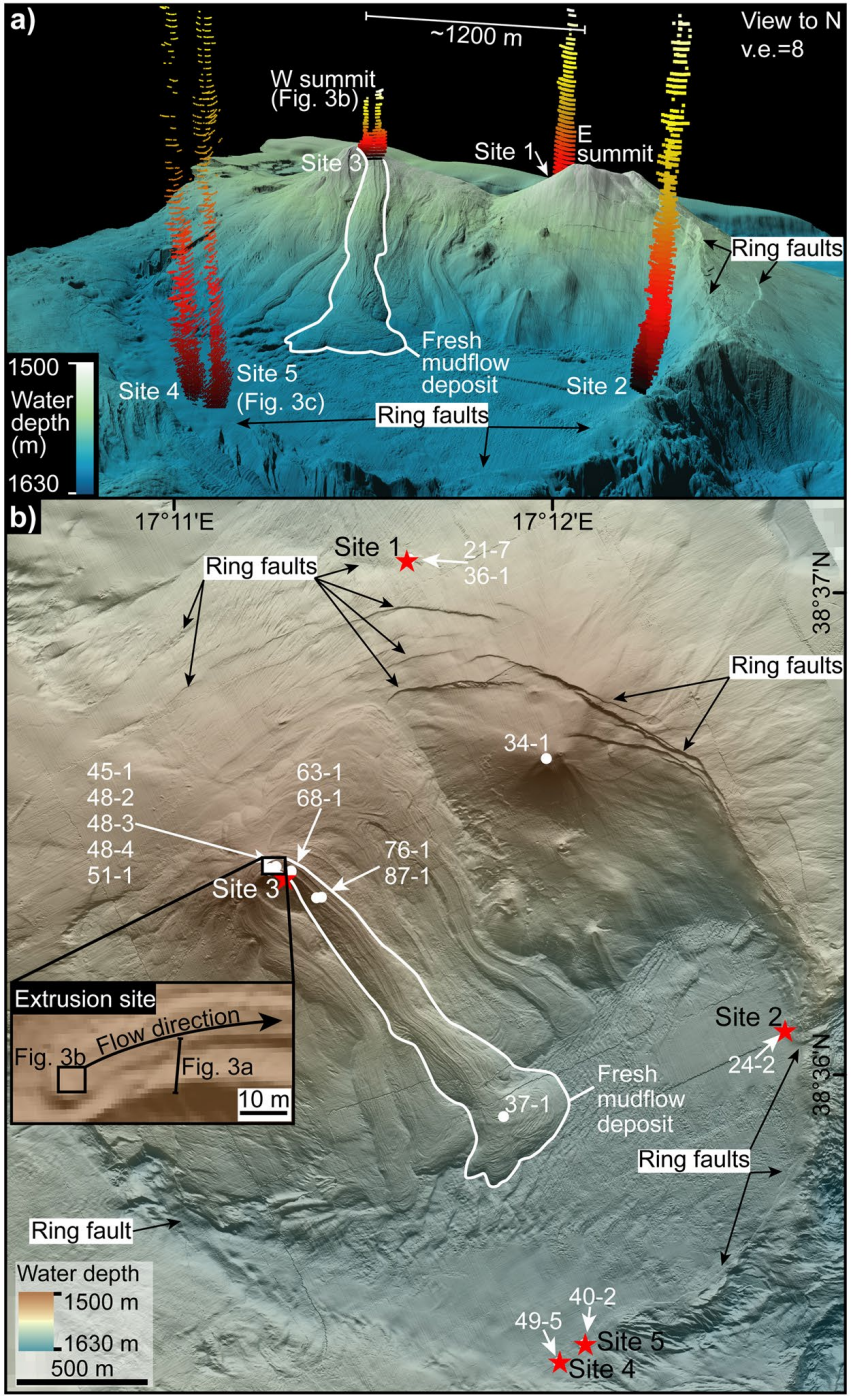
AUV-derived bathymetry (1.6 m grid) of Venere mud volcano (MV) and sampling locations. A fresh mudflow (outlined in white) originating from the W summit extends down to the caldera floor. (**a**) Perspective view of Venere MV (generated in QPS Fledermaus 7.3.2b; www.qps.nl). Note twin cones labelled as E + W summit, each up to 100 m high and ring faults defining a caldera up to 3 km across. Water column gas flares (red to yellow colours; extracted from hydroacoustic data) up to 260 m in height were observed at five sites, along the peripheral ring faults (Sites 1, 2, 4, 5) and near the W summit (Site 3); (**b**) Map-view of Venere MV (see Fig. 1 for extent) with inset of the extrusion site of the fresh mudflow at the W summit (generated in ESRI ArcMap 10.3.1; www.esri.com). Red stars mark the flare origins and white circles indicate sampling locations with white numbers referring to the last two GeoB-identifiers (see Table 1 and supplementary Tables S1 and S2 for full details of all stations). See supplementary Figs S2 and S3 for bathymetry without annotations and an alternative perspective view of a) showing the heights of ring faults.

The seafloor morphology of Venere MV is imaged in detail by AUV-borne swath bathymetric data with a horizontal resolution of up to 1.6 m (Fig. 2). Bathymetric observations reveal that Venere MV consists of an eastern and a western summit ~1,200 m apart (Fig. 2a,b), rising up to 100 m above the surrounding seafloor. The twin cones are cut by concentric sets of inward-dipping scarps with surface slopes of 18–45°, which define a circular feature centered ~700 m SE of the western summit (Fig. 2). The scarps are visible as discontinuous morphological steps that offset seafloor features, with relief of up to 40 m in the east and as low as 1 m in the south and west. No scarps are visible in the northwest. The scarps are interpreted as extensional ring faults, defining a caldera up to 3 km in diameter. Seafloor dips suggest the ring faults may extend to subsurface depths of 1–3 km beneath the mud volcano.

**Figure 3:**
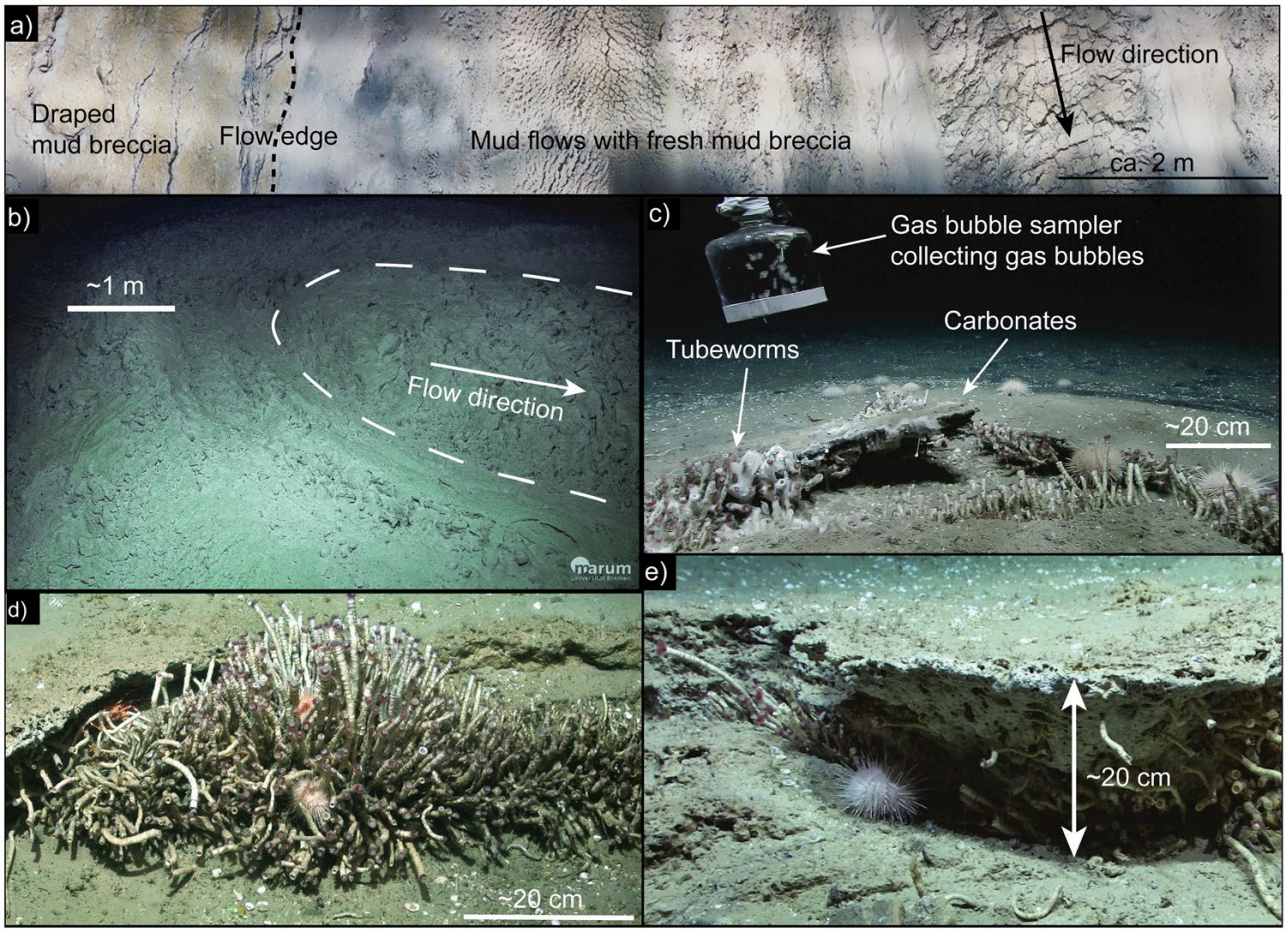
Seafloor photographs. (**a**) ~10 m long transect across fresh (right) vs. older (left) mud breccia flows draped by hemipelagic sediments near the western summit (see inset of Fig. 2b for location); (**b**) western summit of Venere MV showing elevated extrusive centre at the origin of the most recent mudflow (see inset of Fig. 2b for location); (**c**) authigenic carbonate crusts, cold-seep communities and sampling of gas bubbles at a peripheral seep (Site 5); (**d**) tubeworm colony rooting in a fracture of authigenic carbonate crust at a peripheral seep (Site 1); (**e**) cold-seep community and thick authigenic carbonate pavement at a peripheral seep (Site 1).

At the western cone (~1,500 m water depth), elongate mudflow deposits are observed extending for horizontal distances of up to ~1,600 m down the southern flank to the foot of the cone (~1620 m water depth; Fig. 2). This most recent mud breccia outflow deposit was investigated via ROV observations (Fig. 3a,b) and sampling at the western cone (Fig. 2). A fresh mudflow originates from the top of the summit (inset Fig. 2), observed as a slightly elevated horseshoe-shaped feature about 3 m across with a central depression (Fig. 3b). Exposed mud breccia deposits showed a blocky structure that lacked the hemipelagic sedimentary cover visible on adjacent flows (Fig. 3a).

At the eastern cone, bathymetric observations reveal elongate but smooth lobes (Fig. 2), consistent with less recent extrusive activity. This is supported by a sediment core from the eastern summit (GeoB19234-1; Fig. 2), which contained mud breccia draped by several decimetres of hemipelagic sediment (see also Loher *et al*.^27^).

## Water column gas flares linked to ROV seafloor observations

Hydroacoustic surveys conducted 28 times across Venere MV over a 31 day period during M112 revealed temporally variable but generally persistent gas discharge (flares) from five sites: one slightly below the western summit (Site 3) and four peripheral to the MV cones (Sites 1, 2, 4, and 5; Fig. 2). The flares were up to ~260 m high along the peripheral sites but only up to ~50 m high at the summit. The seafloor origins of the four peripheral gas flares (Sites 1, 2, 4, 5) coincide with surface traces of the ring-fault system (Fig. 2).

ROV explorations confirmed gas bubble emissions (Fig. 3c) at each of the sites. They were settled by chemosynthetic organisms and surrounded by mounds and pavements of authigenic carbonate several decimetres thick (Fig. 3c–e). In contrast at Site 3, below the western summit, gas bubble emissions were not encountered during two ROV surveys. On the fresh mudflows and in surrounding areas draped by hemipelagic sediment no macroscopic indications of seepage (carbonates, chemosynthetic communities) were observed (Fig. 3a,b). Thus no seep-related sediment samples could be collected from Site 3.

## Gas and pore water compositions in mud breccia from the western summit

Cores of mud breccia flowing down the flank of the western cone (Table 1, supplementary Table S2) contained rocks clasts to decimetre size in a mud matrix, typically characterized by sediment textures resulting from gas bubbles and pockets giving it a mousse-like appearance. Quantitative degassing of one of the pressure cores (DAPC, GeoB19251-1) yielded 41.3 L of gas from the 11220 cm^3^ of sediment retrieved, which translated to a volumetric gas-sediment ratio of 3.68. In addition to methane (C_1_), heavier hydrocarbons such as ethane (C_2_) and propane (C_3_) were detected in gas samples from the mud breccia, with traces of butane (C_4_) and pentane (C_5_; Table 1). The ratio of methane to higher hydrocarbons (C_1_/C_2+_) in gas samples from the mud breccia ranged between 79 and 107. Methane (CH_4_) constituted 98.5 vol-% of the total gas released from the pressure core and using an average sediment porosity of 0.5 (determined in samples from gravity core GeoB19245-1), the calculated average CH_4_ concentration was 321 mmol/L pore water. This CH_4_ concentration is about 2.7 times higher than the calculated *in-situ* CH_4_ solubility (120 mmol/L pore water; temperature (T) at 2 mbsf = 31.33 °C; salinity (S) = 10 psu; following equations by Tishchenko *et al*.^41^). The stable carbon and hydrogen isotopic composition of methane (δ^13^C-CH_4_; δD-CH_4_) ranged from −36.6 to −41.6‰ V-PDB and from −143.9 to −156.0‰ V-SMOW, respectively (Table 1).

**Table 1:** GeoB-identifier, sampling tool, location (WGS84), and water depth of stations for gravity cores and samples of gas analyses (see Fig. 2 for positions) with molecular hydrocarbon ratios (C_1_/C_2+_, C_2_/C_3_), stable C isotope signatures (δ^13^C, in ‰ V-PDB) of methane (CH_4_), ethane (C_2_H_6_), propane (C_3_H_8_), and stable H isotope signatures (δD, in ‰ V-SMOW) of CH_4_.

GeoB-No.	Sample Type	Lat/N	Lon/E	Site	C_1_/C_2+_	C_2_/C_3_	CH_4_ (δ^13^C)	C_2_H_6_ (δ^13^C)	C_3_H_8_ (δ^13^C)	CH_4_ (δD)
19221-7	GBS	38°37.095′	17°11.602′	Seep, Site 1	*2249	*138	*−48.6	*−21.6	*−12.5	−181.4
19224-2	GBS	38°36.096′	17°12.571′	Seep, Site 2	859	—	−44.7	—	—	−176.0
19249-5	GBS	38°35.429′	17°11.960′	Seep, Site 4	1259	—	−46.7	—	—	−182.6
19240-2	GBS	38°35.458′	17°12.022′	Seep, Site 5	*1360	*140	*−47.3	*−22.3	*−17.7	−181.0
19251-1	DAPC	38°36.452′	17°11.224′	Extrusion site	92	8	−37.4	−27	−21.3	−148.2
19245-1	GC	38°36.455′	17°11.223′	Extrusion site	—	—	−37.4	—	—	−153.8
19263-1	GC	38°36.448′′	17°11.282′	Mudflow	—	—	−41.3	—	—	−155.9
19268-1	DAPC	38°36.450′	17°11.282′	Mudflow	107	22	−41.6	—	—	−156.0
19276-1	GC	38°36.393′	17°11.348′	Mudflow	—	—	−36.6	—	—	−143.9
19287-1	DAPC	38°36.394′	17°11.361′	Mudflow	78	25	−36.6	—	—	−148.7
19237-1	GC	38°35.930′	17°11.828′	Mudflow	—	—	−37.4	—	—	−148.5

*Values from Blumenberg *et al*.^51^.

DAPC = Dynamic Autoclave Piston Corer; GBS = Gas bubble sampler; GC = Gravity corer.

**Figure 4:**
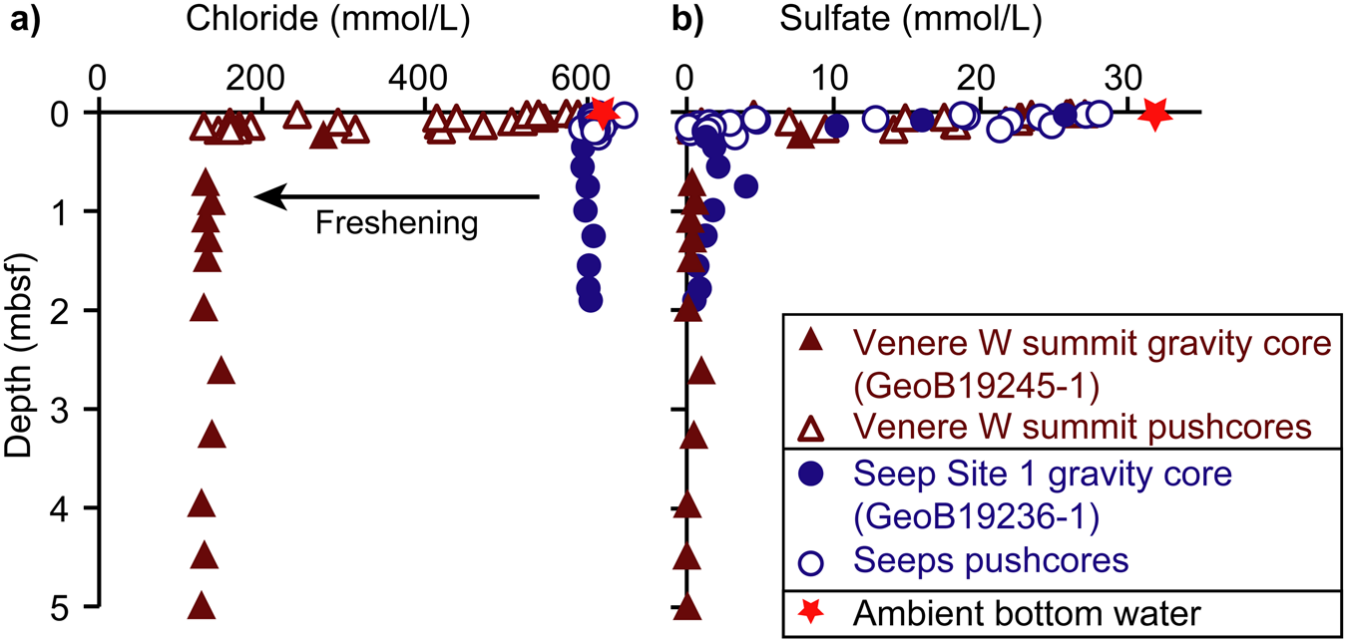
Pore water profiles of chloride (**a**) and sulfate (**b**) from the western summit of Venere MV (triangles) and from peripheral seeps (circles). Locations of gravity cores GeoB19245-1 and GeoB19236-1 in Fig. 2; locations of push cores in supplementary Table S2. Red stars show ambient bottom water concentrations of chloride (618 mmol/L) and sulfate (32 mmol/L), respectively; mbsf = meters below seafloor; data tables are provided in supplementary Tables S3–S13.

Chloride concentrations in fluids extracted from mud breccia outflows at the extrusion site on the western summit, decreased from bottom water values of 618 mmol/L at the sediment surface to 128 mmol/L at 14 cmbsf (Fig. 4a). Gravity core GeoB19245-1 from the extrusion site confirmed that low chlorinity fluids (125 mmol/L at minimum) extended down to 5 mbsf.

## Gas and fluid compositions at peripheral seeps

Pore fluid samples in gravity cores and push cores obtained from peripheral seeps (Sites 1, 2, 4 and 5, Fig. 2) showed chloride concentrations within 4% of bottom water values to depths up to 2 mbsf (Fig. 4a). All peripheral seeps hosted abundant chemosynthetic macrofauna and authigenic carbonate crusts (Fig. 3c–e). These are indicative of a shallow zone of anaerobic methane oxidation, consistent with measured steep sulfate gradients and sulfate-methane transitions at depths of <25 cmbsf (Fig. 4b). The discharged gas at peripheral seeps was composed primarily of methane but also contained ethane (C_2_) and propane (C_3_). Gas bubbles sampled at the peripheral seeps had C_1_/C_2+_ ratios of 859–2249, with δ^13^C-CH_4_ and δD-CH_4_ ranging from −44.7 to −48.6‰ V-PDB and from −176.0‰ to −182.6 V-SMOW, respectively (Table 1).

## Sediment temperatures at the extrusion site and the peripheral seeps versus gas hydrate stability

In the mud breccia at the extrusion site (Fig. 2) the maximum temperature, measured at 40 cm above the tip of a 5 m gravity core barrel (GeoB19248-2), was 26.8 °C. This is 13 °C higher than average bottom water temperatures. In addition, sediment temperatures above 20 °C were repeatedly measured below 0.5 mbsf (meters below seafloor). The average of linear temperature gradients calculated for the mudflow at the extrusion site amounted to 8.76 °C/m (supplementary Table S1). Regardless of their crystal structure specific stability, gas hydrates would only be stable within 30 cm of the seafloor (supplementary Fig. S1).

Averages of linear temperature gradients close to the gas emission sites at the peripheral seeps ranged from 0.15–0.20 °C/m (supplement Table S1). Using these gradients, the calculated base of the gas hydrate stability at the seeps is located 8–10 mbsf (supplement Fig. S1). Seafloor sampling of some of the seeps with the ROV resulted in the release of flake-like white particles (several mm in diameter) that rose into the water column, suggesting the subsurface presence of small amounts of gas hydrates, associated with seepage along the ring faults.

## Deep fluid sources

The recent mud breccia outflows are rich in gas. *In-situ* methane concentrations exceeding CH_4_ solubility by up to 2.7 times as determined for pressure cores imply that a large fraction of the pore space was still occupied by gaseous methane. High gas contents are also consistent with the mousse-like sediment textures observed in unpressurized cores. Using the classification by Whiticar^42^ (supplementary Fig. S4a,b), the molecular composition of hydrocarbons and isotopic compositions of methane (Table 1), point to a thermogenic origin for the hydrocarbons transported within the mud breccia of Venere MV.

Formation temperatures range from ~70 °C for the early thermogenic hydrocarbons to upwards of 150 °C for late thermogenic, methane-rich gas production systems^43–45^. Venere MV lies midway between geothermal gradients as low as 0.013 °C/m offshore and 0.024–0.030 °C/m onshore^46^. Using an average geothermal gradient of 0.020 °C/m at the location of Venere MV, and thermogenic temperatures of 70–150 °C, the presence of thermogenic hydrocarbons in the mud breccia outflows suggests a source located at least 3.5 km and potentially >7.5 km below seafloor. This depth range corresponds to empirically determined burial depths for source rocks within the thermogenic window (e.g. Quigley and Mackenzie^44^). This does not preclude the occurrence of a pressurized reservoir trapping these gases at shallower depths (i.e. cooler temperatures) below Venere MV.

Mud breccia at the western summit of Venere MV is also characterized by pore water freshening (Fig. 4a). The magnitude of chloride depletion and the abrupt reduction within the uppermost 10 to 20 cmbsf document an upward advective aqueous component in the extruding mud. The continuously low chloride concentrations at depth indicate that the freshening is unlikely to be associated with gas hydrate decomposition, which would instead cause discrete low-chloride spikes (e.g. Torres *et al*.^47^). An absence of gas hydrates at the western summit of Venere MV is consistent with measured sediment temperatures, which shift mud breccia at >30 cmbsf outside the gas hydrate stability zone (supplementary Fig. S1). Instead, the pore water profiles are consistent with extrusion of mud breccia containing fresh fluids sourced at depth, likely driven by mineral dehydration reactions, such as the transformation of smectite to illite, which takes place at temperatures between ~60 and 150 °C^48^. Pore-water freshening has also been observed in MVs on the Mediterranean Ridge, where the freshening has been attributed to mineral dehydration during smectite-illite transition at depths of 3.5–7 km^21^. At Venere MV, assuming a regional geothermal gradient of 0.02 °C/m (as above), the temperature range of 60–150 °C translates to fluid generation at 3–7.5 km, in accordance with the formation depths required by the thermogenic hydrocarbons.

These results indicate that formation of thermogenic hydrocarbons and water release by mineral dehydration reactions led to the generation of the fluids that are associated to the mud breccia at Venere MV. These processes require temperatures ≥60 °C that, at the location of Venere MV, indicate source depths exceeding 3 km. These depths lie well below those of the inferred depth range of Messinian deposits interpreted to occur in the Crotone forearc basin (Fig. 1d^32^). On the CAP, MV sources have previously been inferred to underlie the forearc-basin sediments based on mud breccia compositions containing fossils and clasts derived from strata as old as Cretaceous^2,38,39^. It is unknown if Messinian deposits in the Crotone forearc basins contain evaporites, however, at Venere MV the mud breccia containing strongly freshened pore waters offers no evidence of interactions with Messinian evaporites during their rise from depth. One possibility is that Messinian deposits, if present in the sedimentary succession below the location of Venere MV, did not contain evaporites. This possibility is in accordance with other studies, which found that MVs in the eastern Mediterranean region preferably developed at sites where Messinian deposits are thin or absent^18,25,32,36^ or cut by deep-seated faults acting as fluid-migration pathways^18,24^. Another option is that, over time, fluid migration through the plumbing system beneath Venere MV has removed any Messinian evaporites.

## Upward-branching plumbing system

**Figure 5:**
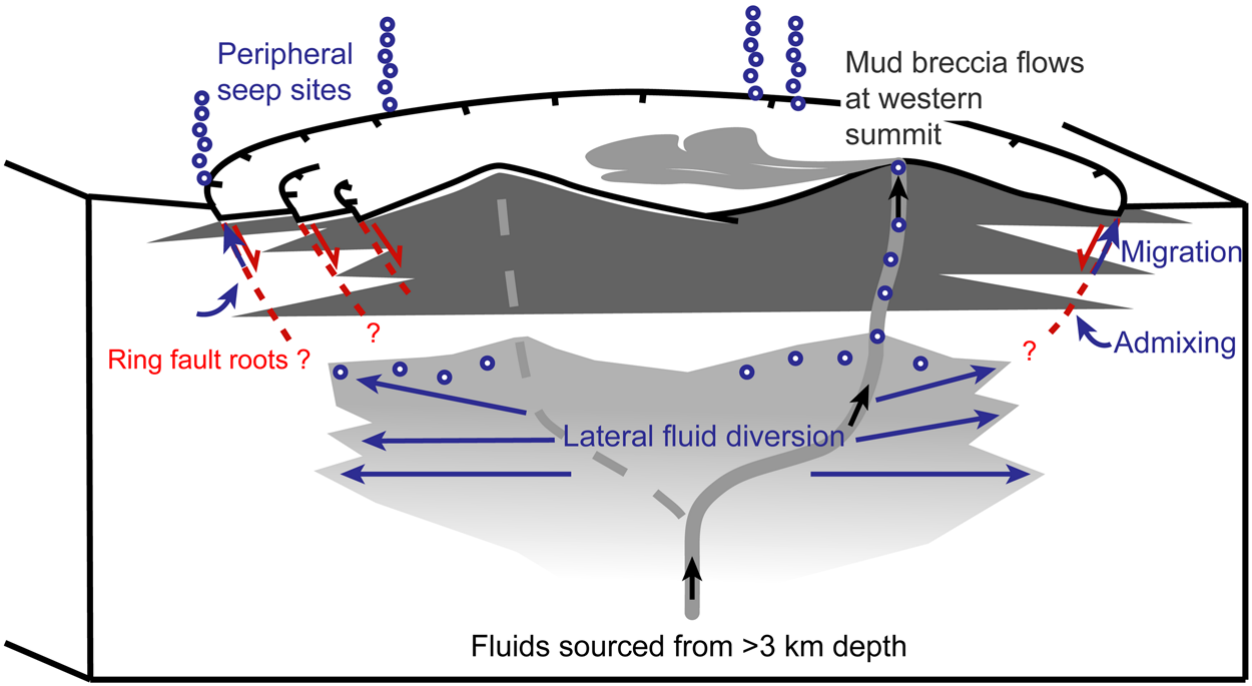
Conceptual model of the upward-branching plumbing system. Gas-rich mud breccia supply (black arrows) through active conduit (solid light grey line) of main plumbing system from depth to the summit of the western MV cone; the stippled light grey line represents a presumably inactive conduit at the eastern cone, dark grey wedges represent buried mud breccia deposits and red stippled lines represent ring faults. Gas diverted laterally from the main conduit (long blue arrows in light grey area) migrates upward (short blue arrows) and may mix with gas of shallow origin (indicated by curved blue arrows). Gas discharge occurs at peripheral seeps (blue circles represent free gas).

Gas seepage and outflow of fluid-rich muds along MV ring-faults is known from studies on land and typically characterizes quiescent phases of MVs^8,49^. However, the processes leading to the co-occurrence of quiescent-type activity and active mud breccia extrusions are not well understood. Based on observations of the prevailing seafloor morphologies and processes, and analyses of pore fluid (gas and water) compositions of samples from Venere MV, a conceptual model of its plumbing system is proposed that inter-relates extrusive processes, ring-faulting, fluid seepage and seafloor ecosystems (Fig. 5).

The rough surface structure of exposed mud breccia observed at and below the extrusion site (Fig. 3a,b) together with the lack of sedimentary drape on the most recent mudflow indicate that these deposits are fresh. The lack of cold seep features (carbonate, chemosynthetic communities) is also consistent with a dynamic sedimentary environment. The high sediment temperatures measured within the extrusion site are also consistent with recent or even on-going extrusion, as are pore water profiles with steep sulfate gradients documenting the advection of sulfate-depleted mud (Fig. 4b). Calculations of ascent rates of the mud breccia, based on the observed clast sizes (up to 10 cm), provide an order of magnitude estimate of 1.7*10^−5^ to 1.7*10^−4^ m/s (see supplementary material for calculations). The resulting estimate of the mud breccia flux through the conduit of 3800 to 38000 m^3^/year fits well with published rates of 5000 to 47000 m^3^/year for mud breccia extrusions at Venere MV over the last ~882 years^27^.

The deeply-rooting conduit inferred to rise beneath the western summit is encircled by inward-dipping ring-faults. Gas discharge at peripheral sites indicates that fluids are migrating along the faults. The peripheral seeps of Venere MV are so far the only sites on the CAP from which extensive chemosynthesis-based communities associated with gas emissions and authigenic carbonate deposits have been documented. These observations indicate a long-term upward supply of dissolved methane at Venere MV. The presence of decimetre-thick authigenic carbonates in particular indicate persistent gas seepage over timescales of thousands of years^27^. Considering the timing of mud breccia extrusions from the western cone (recent to on-going) and the quiescent-type gas release along the ring faults (long-term and on-going) it is argued that both processes have been occurring coevally.

The development of the caldera at Venere MV has been proposed to result from the long-term extrusive activity involving material withdrawal from the subsurface and thus linking active mud volcanism to caldera subsidence involving ring-fault movements^27^. Faults bordering MV edifices are a known features of submarine MVs (e.g. Paull *et al*.^50^). In a deep-marine environment, however, the high-resolution bathymetric dataset presented here reveals in unprecedented detail the seafloor expression of a ring-fault bounded caldera with sites of active fluid release along it. The stable carbon and hydrogen isotopic signatures of methane collected at the peripheral seeps (Table 1) indicate a thermogenic methane source using the classification by Whiticar^42^ (supplementary Fig. S4a). This is in agreement with results obtained by Blumenberg *et al*.^51^ who documented the presence of methane, ethane, and propane in gas from the peripheral seeps (Table 1), and attributed a thermogenic origin for this gas. C_1_/C_2+_ ratios determined for the gas, however, are higher than expected for hydrocarbons from an exclusive thermogenic source (see also supplementary Fig. S4b). Several post-genetic processes can account for the molecular and isotopic relationships of hydrocarbons at the peripheral seeps: admixture of shallow microbial methane during fluid ascent; secondary microbial methane generation leading to drier gas with relatively more negative δ^13^C-CH_4_ values^52,53^; and segregation effects during gas transport through porous, aqueous sediments^54^.

Nevertheless, the gas discharging at the periphery of Venere MV clearly contains thermogenic hydrocarbons (C_2_ and C_3_; Table 1) as the mud breccia extruded through the main conduit at the western summit. It is therefore argued that the thermogenic fraction of the gas released at the peripheral seeps branched upward and laterally away from the main MV conduit (Fig. 5). Furthermore, the ring faults act as natural migration pathways for gas but not for mud breccia. It appears that the overpressured gas undergoes upward divergence as it migrates through faults and microfractures of the ring-fault system. Similar lateral fluid migration has been previously proposed to occur at MVs, at depths where overpressured fluids experience lower surrounding pressures^4^. As shown for the sediment-hosted hydrothermal system of the Lusi site in Indonesia, divergent fluid migration along faults of collapsing calderas takes more time than focused flow along the main conduit^55^. This may result in changes in the composition of vented gases. At Venere MV, the longer distances and possibly slower flow of gas along the ring-fault system may have promoted the admixing of shallow microbial methane and contributed to changes in the gas molecular composition (Fig. 5). Both admixture and migration processes typically contribute to an increase in the C_1_/C_2+_ ratio and manifest through more negative δ^13^C-CH_4_ values, as observed at gas emitted at the peripheral seeps (Table 1).

Mud volcanism in general is driven by the cyclic build-up of subsurface overpressure triggering the material release^56^. In the proposed model (Fig. 5), the ring faults are argued to respond to variations in pore pressures linked to fluid pulses from the deeper plumbing system. During phases of high pore fluid pressures the ring faults facilitate the diversion of an overpressured gas phase to the periphery of the MV. In turn, variations in pore pressures may facilitate movement on the ring-faults (e.g. leading to caldera collapse). For most offshore MVs, their dynamics and variability of activity remain unknown. More quantitative information on fluid and sediment amounts involved is essential to constrain the global contribution of MVs to material fluxes at geosphere-hydrosphere interfaces. Advances in hydroacoustic and visual imaging technologies and the use of AUVs broaden the capabilities in identifying and investigating seafloor seep sites expressing various forms of activity.

## Conclusion

The first visual evidence of active gas emissions on the CAP is reported. Among over 50 MVs, only Venere MV was encountered to show activity during systematic hydroacoustic investigations of the CAP in November– December 2014. Contrasting to previous assumptions on fluid dynamics associated to mud volcanism, this study reveals that two fluid domains can co-exist: 1) mud breccia outflows, extrusion of thermogenic hydrocarbons, and freshened pore waters at a summit site; and 2) long term advective seepage of methane fuelling cold-seep communities at sites along peripheral ring-faults. Mud breccia extrusion, gas release along peripheral seeps, and caldera ring-faulting appear to be linked and sustained by persistently high pore fluid pressures. Fluids discharging with mud breccia at the summit are generated by thermogenic organic matter degradation and mineral dehydration reactions occurring at depths of >3 km. This formation depth implies that fluid sources are located below the Messinian to Recent sedimentary infilling of the Crotone forearc basin. Freshened pore waters indicated that Messinian evaporites are either not present or have been removed by fluid migration from below Venere MV.

Considering the presence of thermogenic hydrocarbons in both fluid domains, it is inferred that seepage at Venere MV involves an upward-branching plumbing system and that overpressured gas diverted laterally from the main MV conduit migrates upward and mixes with gas of shallow origin. As a result, chemosynthesis-based communities that rely on persistent fluid supply and thick authigenic carbonates are limited to the peripheral seeps along the ring faults of the caldera. An upward-branching plumbing system that experiences high subsurface pore pressures can explain how both mud breccia discharge and quiescent-type seepage co-exist at an individual MV. Such a mechanism may allow for mud breccia discharge to be sustained over timescales exceeding the short-lived eruptions typically associated with active mud volcanism. Furthermore, the upward-branching fluid migration pathways exert an important control on the distribution of seepage-dependent chemosynthesis-based oases of life around submarine MVs.

## Methods

### Hydroacoustics

Multi-disciplinary investigations of Venere MV were undertaken in 2014 and 2016 during R/V METEOR cruise M112^26^ and R/V POSEIDON cruise POS499^57^. Swath bathymetry data were collected using two Kongsberg multibeam systems, a ship-borne EM122 (12 kHz) in 2014 and an EM2040 (300 kHz) carried by the autonomous underwater vehicle (AUV) MARUM-SEAL 5000 in 2014 and 2016. Bathymetric datasets were processed with MB-System^58^. Ship-based data was gridded to 25 m lateral and meter-scale vertical resolution and AUV-based data to 1.6 m lateral and 0.1 m vertical resolution.

### Sediment samples and temperature

Sediment cores up to 5 m long were acquired using a conventional gravity corer and the Dynamic Autoclave Piston Corer (DAPC), the latter allowing for retrieval of cores under *in-situ* pressure^59^. Seafloor observations, sediment sampling with push cores (30 cm long) and collection of gas bubbles from the water column were conducted using the remotely operated vehicle (ROV) MARUM QUEST 4000 m during M112^26^. *In-situ* sediment temperatures were measured using miniaturized temperature data loggers (MTLs, ANTARES Datensystem GmbH), mounted as an array of 6 sensors on a 5 m gravity core barrel or with a 60 cm long probe (T-stick; 8 sensors), inserted vertically into the sediment using the ROV^60^.

### Pore water and gas analysis

Pore fluids were extracted from gravity cores and push cores using rhizon samplers. Sulphate and chloride concentrations were determined by ion-chromatography (“882 Compact IC plus 1” by Metrohm on a “Metrosep A Supp 5” column) with an analytical error below 0.4% for both anions. Hydrocarbon data was generated from sediment by headspace technique^61^; from gas bubble sampling under *in-situ* pressures over the seeps using the ROV (Fig. 3c); and from the DAPC^59^. Methane *ex situ* concentrations and hydrocarbon compositions were analysed during M112 by gas chromatography^15^. Stable carbon and hydrogen isotope ratios (^13^C/^12^C; ^2^H/^1^H) of methane (CH_4_) were determined by gas chromatography coupled to isotope ratio mass spectrometry (GC-IRMS) at MARUM. Repeated analyses of standards gave a reproducibility for C_1_/C_2+_ ratios, stable carbon, and hydrogen isotope ratios of ≤2%, 0.5% and 1%, respectively. Values of ethane (C_2_) and propane (C_3_) for GeoB19251-1 were analyzed by GEO-data (Environmental-Laboratory in Garbsen, Germany).

### Gas hydrate stability calculation

The gas hydrate phase boundary (supplementary Fig. S1) was calculated with the program HWHYD^62^. Calculations were performed for gas compositions obtained from mud breccia (extrusion site at western summit; Fig. 2b) and gas bubbles (peripheral seeps, Sites 1, 2, 4 and 5). Salinities of 10 PSU (freshened pore water values in mud breccia) and 38.5 PSU (bottom water values at peripheral seeps, Sites 1, 2, 4 and 5) were used as input. Conductivity, temperature, and depth (CTD) measurements were recorded above each site by a Sea Bird Electronics SBE9plus probe^26^. Temperature gradients (supplement Table S1) were calculated from temperatures in shallow sediments at the seep sites. Averaged gradients were used at sites where repeated temperature measurements were carried out. The gradients were extrapolated to determine the base of the gas hydrate stability zone (BGHSZ) at the seeps (supplement Fig. S1).

### Data availability

Relevant data will be made available on PANGAEA® (www.pangaea.de).

## Electronic supplementary material

Dataset 1
