# Supplementary material for: Mud extrusion and ring-fault gas seepage – upward branching fluid discharge at a deep-sea mud volcano
Source: Sci Rep. 2018 Apr 19;8:6275. doi: 10.1038/s41598-018-24689-1 (PMC5908856; doi:10.1038/s41598-018-24689-1)
Supplement: Supplementary file 1 — Dataset 1 [file 41598_2018_24689_MOESM1_ESM.doc]

Supporting Information

# Mud extrusion and ring-fault gas seepage – upward branching fluid discharge at a deep-sea mud volcano

**M. Loher^1^,*, T. Pape^1^, Y. Marcon^1^, M. Römer^1^, P. Wintersteller^1^, D. Praeg^2^, M. Torres^3^, H. Sahling^1^, G. Bohrmann^1^**

^1^*MARUM – Center for Marine Environmental Sciences and Department of Geosciences at University of Bremen, Klagenfurter Str., 28359 Bremen, Germany*

^2^*OGS (Istituto Nazionale di Oceanografia e di Geofisica Sperimentale), Borgo Grotta Gigante 42/c, Sgonico, 34010 Trieste, Italy; present address: Institute of Petroleum and Natural Resources, PUCRS, Av. Ipiranga, 6681, 90619-900 Porto Alegre, RS, Brazil*

^3^*College of Earth, Ocean, and Atmospheric Sciences, Oregon State University, 104 CEOAS Administration Building, Corvallis, OR 97331-5503, USA*

**corresponding author e-mail:* [*mloher@marum.de*](mailto:mloher@marum.de)

We provide information on how the mud breccia ascent rate and volume flux was calculated (text). Further we provide four figures (Fig. S1-S4) as supplement to the main article. Two tables (Tables S1-S2) containing information on all sampling locations (sediments, pore water, temperature gradients) and eleven tables (Table S3-S13) containing information on pore water data used in the figures and main text.

All temperature measurements will be made available via Pangaea–Data publisher for Earth & Environmental Science ([www.pangaea.de](http://www.pangaea.de/)).

**Calculations to estimate mud breccia ascent rates and volume fluxes.** Ascent rates of mud breccia through circular, pipe-like conduits have been estimated based on the settling velocity (v) of mud breccia clasts by using Stoke’s law (assuming laminar flow, spherical particles, homogeneous materials, smooth surfaces, and no interference of the particles) following studies by Kopf and Behrman^n 1^ and Collignon, et al^. 2^:


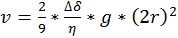
 (1)

where ∆δ represents the density difference between the clast and the mud matrix, η is the fluid dynamic viscosity, g is the gravitational acceleration, r is the radius of the particle. As density for the sediment lithologies present in mud breccia of Venere MV (carbonates, sandstones, friable shale, and marls) ^3^, a density of 2600 kg/m^3^ is assumed and for the mud matrix a density of 1815 kg/m^3^ results (depending on a porosity of 0.5 with a pore-water density of 1030 kg/m^3^). The maximum size for the clast diameter was found to be approximately 10 cm. For the viscosity, values from Kopf and Behrman^n 1^ of 10^6^ Pa s are used (who determined this value for mud breccia with a mousse-like sediment texture) and of 10^5^ Pa s following Collignon, et al^. 2^ are used to constrain the minimum (1.7*10^-5^ m/s) and maximum (1.7*10^-4^ m/s) values for the ascent velocity. Formula (1) can be modified to determine the volume mud breccia flux by adding a term for the cross-sectional area of the feeder channel ^1^: R^2^*π, where R is the radius of the channel estimated to be 1.5 m as at the extrusion site at the summit of Venere MV (Fig. 3b). Such a radius is similar to those estimated for other MV conduits of 1.4 to 1.7 m for the Lusi structure ^2^ or diameters of 2 to 3 m reported from several studies of terrestrial MVs as well as theoretical considerations and calculations from Mediterranean Ridge MVs ^1,4^. The resulting minimum to maximum volume flux of mud breccia are approximately 3800 to 38000 m^3^/year, which are the same order of magnitude as values of 5000 to 47000 m^3^/year determined independently for Venere MV by Loher, et al^. 3^.

## Fig. S1

| 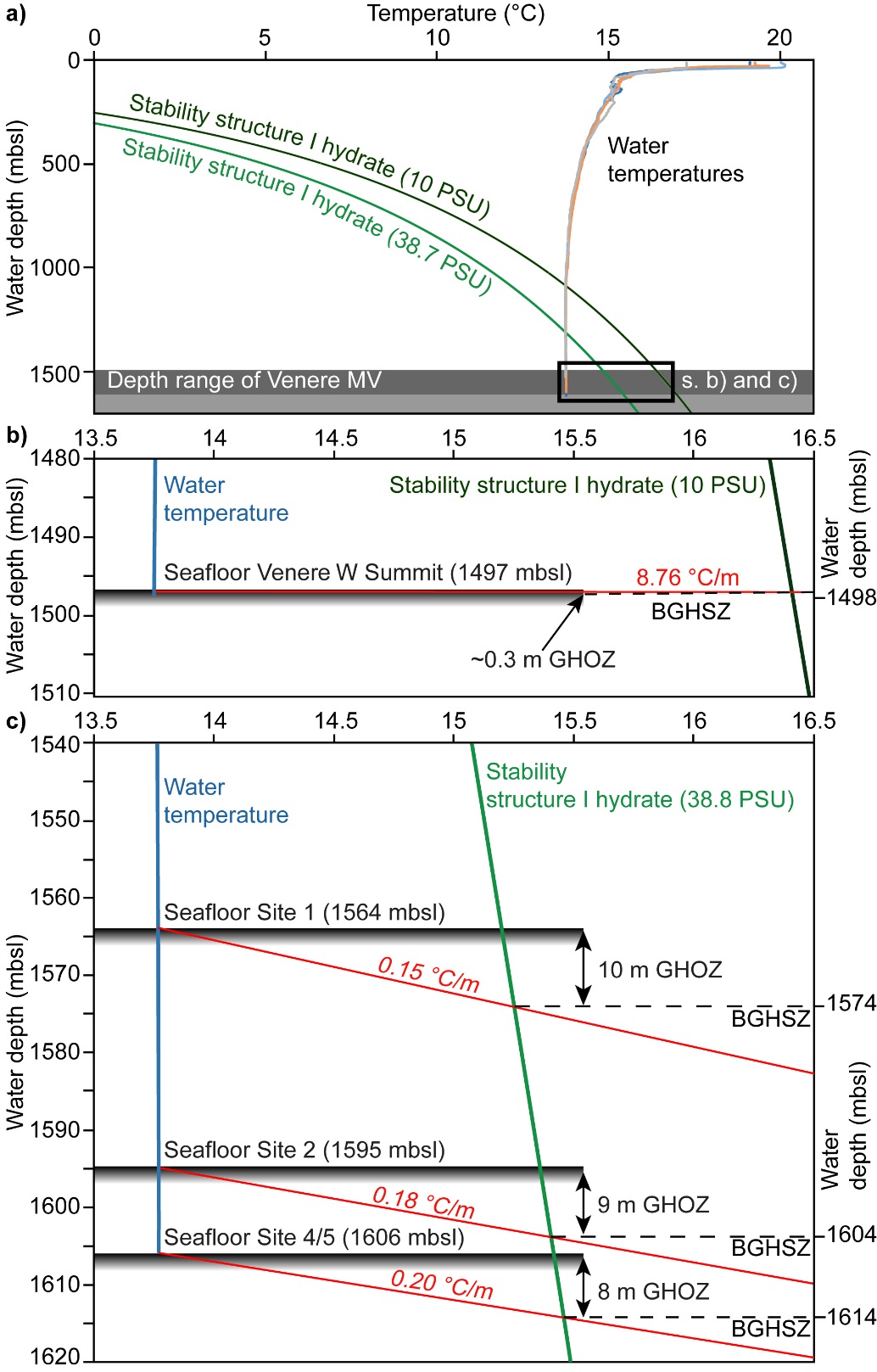 |
| --- |
| Figure S1. a: Calculated phase boundaries for structure I hydrates at Venere MV; b+c: enlargements of diagram shown in (a) with black stippled lines marking the base of the gas hydrate stability zone (BGHSZ) to define the gas hydrate occurrence zone (GHOZ), b) shows averaged, linear geothermal gradient at the western summit of Venere MV, note the steep temperature gradient indicating that gas hydrates are unlikely to be present at the summit (~0.3 m for GHOZ), c) shows averaged, linear geothermal gradients for peripheral seeps (Sites 1, 2 and 4/5); mbsl = meters below sea level. |

## Fig. S2

| 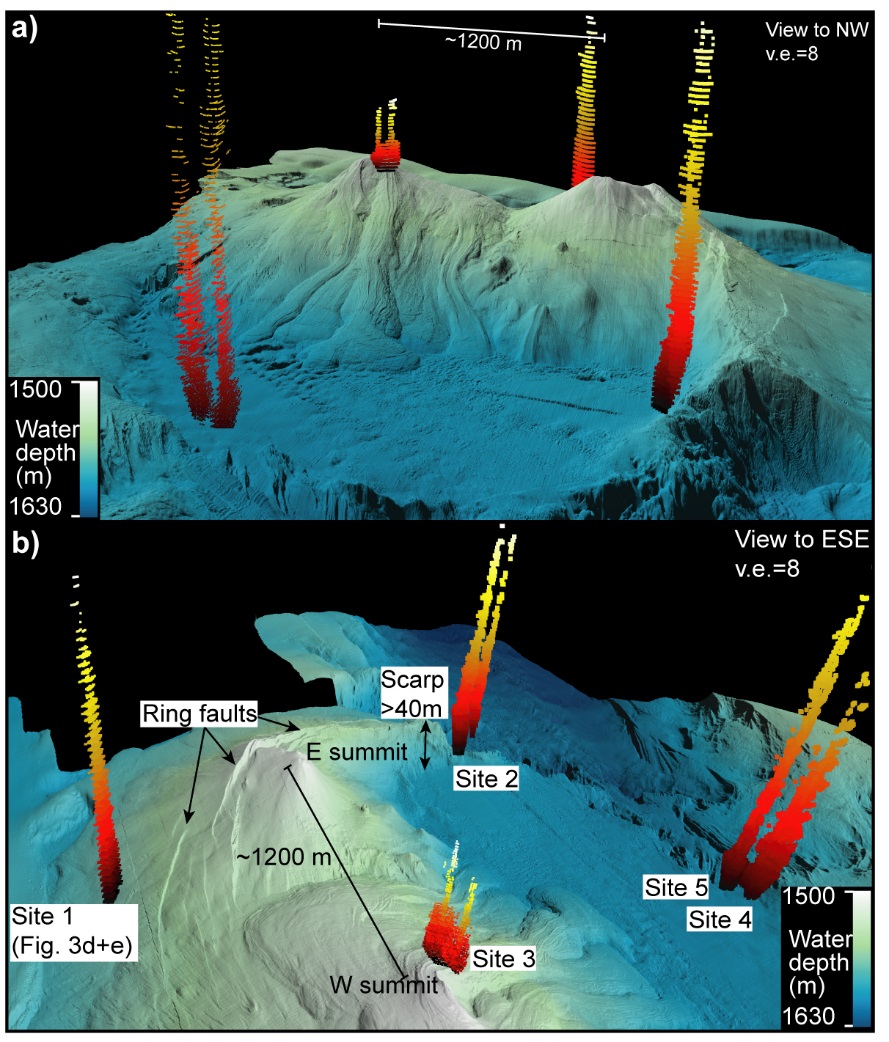 |
| --- |
| Fig. S2. a: Same as Fig. 2a in main manuscript but without annotations for clearer view; b: Same as a) but view to ESE with better perspective of ring faults on northeastern edge, note scarp height of >40 m at E edge of caldera; perspective views generated in QPS Fledermaus 7.3.2b (www.qps.nl). |

## Fig. S3

| 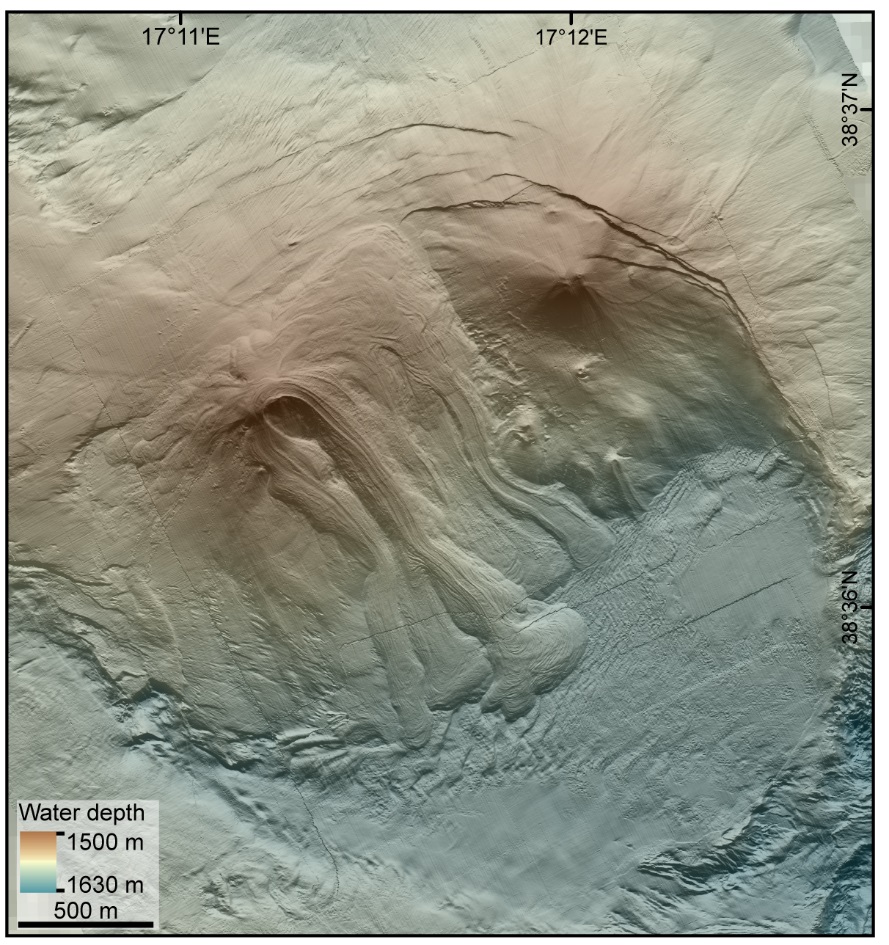 | |
| --- | --- |
| Fig. S3.: Same as Fig. 2b) of main manuscript but without annotations for clearer view. Map generated in ESRI ArcMap 10.3.1 (www.esri.com). |  |

## Fig. S4

| 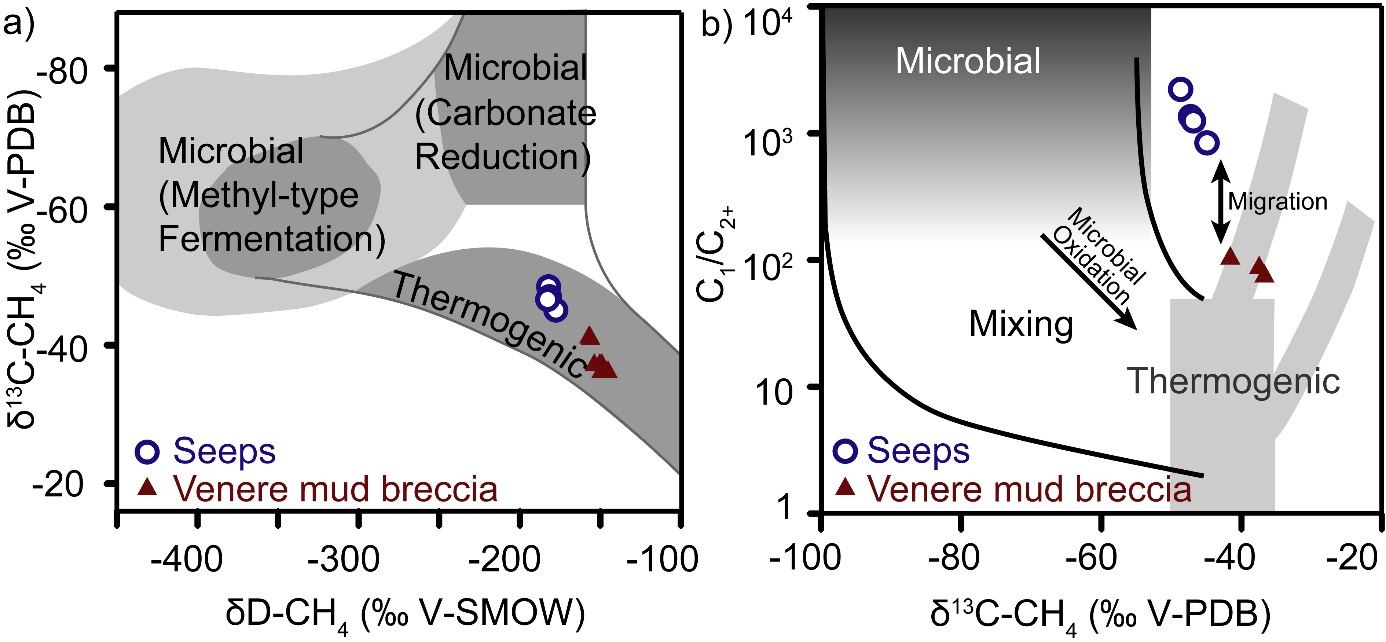 |
| --- |
| Figure S4. Molecular hydrocarbon and methane isotope composition of samples from Venere MV summit (extrusion site) and mudflow (filled triangles) and peripheral seeps (circles). a: Carbon-hydrogen diagram for classification of microbial and thermogenic methane modified after Whitica^r 5^; b: Diagram of C_1_/C_2+_ vs. δ^13^C-CH_4_ (after Bernard, et al^. 6^), showing the thermogenic, microbial, and mixed domains as proposed by Whitica^r 5^. |

**Table S1.** GeoB-identifier, sampling tool, location (WGS84), and water depth of stations for temperature measurements and calculated linear temperature gradients (T-stick = 60 cm long temperature probe deployed by ROV; GC-MTL = Gravity corer equipped with miniaturized temperature loggers).

| GeoB-No. | Tool | Lat / N | Lon / E | Water depth (m) | Site | Temperature gradient |
| --- | --- | --- | --- | --- | --- | --- |
|
| 19202-2 | T-stick | 38°37.084 | 17°11.593 | 1567 | Seep, Site 1 | 0.01 °C/m |
| 19221-3 | T-stick | 38°37.095 | 17°11.602 | 1568 | Seep, Site 1 | 0.16 °C/m |
| 19221-5 | T-stick | 38°37.094 | 17°11.600 | 1567 | Seep, Site 1 | 0.20 °C/m |
| 19230-3 | T-stick | 38°37.095 | 17°11.609 | 1568 | Seep, Site 1 | 0.31 °C/m |
| 19230-10 | T-stick | 38°37.102 | 17°11.630 | 1567 | Seep, Site 1 | 0.06 °C/m |
| 19267-7 | T-stick | 38°36.100 | 17°12.564 | 1596 | Seep, Site 2 | 0.18 °C/m |
| 19240-4 | T-stick | 38°35.457 | 17°12.021 | 1607 | Seep, Site 5 | 0.18 °C/m |
| 19249-3 | T-stick | 38°35.454 | 17°12.020 | 1607 | Seep, Site 5 | 0.22 °C/m |
| 19242-4 | T-stick | 38°36.453 | 17°11.223 | 1499 | Extrusion site | 14.92 °C/m |
| 19242-7 | T-stick | 38°36.453 | 17°11.223 | 1499 | Extrusion site | 15.39 °C/m |
| 19242-13 | T-stick | 38°36.454 | 17°11.225 | 1499 | Extrusion site | 14.66 °C/m |
| 19242-16 | T-stick | 38°36.457 | 17°11.226 | 1500 | Extrusion site | 15.54 °C/m |
| 19248-2 | GC-MTL | 38°36.452 | 17°11.221 | 1500 | Extrusion site | 2.73 °C/m |
| 19248-3 | GC-MTL | 38°36.452 | 17°11.223 | 1498 | Extrusion site | 2.51 °C/m |
| 19248-4 | GC-MTL | 38°36.454 | 17°11.226 | 1495 | Extrusion site | 1.96 °C/m |

**Table S2.** GeoB-identifier, sampling tool, location (WGS84), and water depth of stations for sediment sampling used for pore water extraction (PC = Push core taken by ROV; GC = Gravity core).

| GeoB-No. | Tool | Lat / N | Lon / E | Water depth (m) | Site | Data in supplement |
| --- | --- | --- | --- | --- | --- | --- |
|
| 19230-5 | PC | 38°37.095' | 17°11.609' | 1567 | Seep, Site 1 | Table S3 |
| 19236-1 | GC | 38°37.094' | 17°11.605' | 1557 | Seep, Site 1 | Table S4 |
| 19267-4 | PC | 38°36.100' | 17°12.563' | 1596 | Seep, Site 2 | Table S5 |
| 19249-7 | PC | 38°35.429' | 17°11.959' | 1606 | Seep, Site 4 | Table S6 |
| 19240-10 | PC | 38°35.486' | 17°12.074' | 1608 | Seep, Site 5 | Table S7 |
| 19242-6 | PC | 38°36.453' | 17°11.223' | 1499 | Extrusion site | Table S8 |
| 19242-10 | PC | 38°36.463' | 17°11.247' | 1502 | Extrusion site | Table S9 |
| 19242-11 | PC | 38°36.460' | 17°11.243' | 1502 | Extrusion site | Table S10 |
| 19242-14 | PC | 38°36.454' | 17°11.225' | 1499 | Extrusion site | Table S11 |
| 19242-17 | PC | 38°36.456' | 17°11.227' | 1500 | Extrusion site | Table S12 |
| 19245-1 | GC | 38°36.455' | 17°11.223' | 1496 | Extrusion site | Table S13 |

**Table S3**: Sediment depth indication, chloride and sulfate concentrations of pore water analyses of GeoB19230-5.

| Depth / cmbsf | Chloride / mmol/L | Sulfate / mmol/L |
| --- | --- | --- |
| 4.5 | 603.2 | 19.1 |
| 8.5 | 623.0 | 4.8 |
| 12.5 | 607.5 | 0.8 |
| 16.5 | 592.9 | 0.9 |
| 20.5 | 612.9 | 0.3 |
| 24.5 | 612.5 | 3.3 |

**Table S4**: Sediment depth indication, chloride and sulfate concentrations of pore water analyses of GeoB19236-1.

| Depth / cmbsf | Chloride / mmol/L | Sulfate / mmol/L |
| --- | --- | --- |
| 3 | 601.6 | 25.7 |
| 8 | 601.0 | 16.0 |
| 14 | 600.8 | 10.2 |
| 25 | 601.2 | 1.3 |
| 35 | 593.9 | 1.9 |
| 55 | 593.6 | 2.2 |
| 75 | 599.7 | 4.0 |
| 99 | 597.2 | 1.8 |
| 125 | 607.1 | 1.3 |
| 155 | 601.6 | 0.7 |
| 178 | 600.4 | 0.9 |
| 190 | 603.2 | 0.5 |

**Table S5**: Sediment depth indication, chloride and sulfate concentrations of pore water analyses of GeoB19267-4.

| Depth / cmbsf | Chloride / mmol/L | Sulfate / mmol/L |
| --- | --- | --- |
| 3 | 613.9 | 27.13 |
| 7 | 610.8 | 12.83 |
| 11 | 615.1 | 1.52 |
| 15 | 618.8 | 0.13 |
| 19 | 610.6 | 1.27 |

**Table S6**: Sediment depth indication, chloride and sulfate concentrations of pore water analyses of GeoB19249-7.

| Depth / cmbsf | Chloride / mmol/L | Sulfate / mmol/L |
| --- | --- | --- |
| 3 | 644.7 | 18.7 |
| 7 | 604.6 | 4.6 |
| 11 | 605.7 | 2.9 |
| 15 | 607.1 | 1.8 |
| 19 | 607.6 | 1.4 |

**Table S7**: Sediment depth indication, chloride and sulfate concentrations of pore water analyses of GeoB19240-10.

| Depth / cmbsf | Chloride / mmol/L | Sulfate / mmol/L |
| --- | --- | --- |
| 1.5 | 613.0 | 28.1 |
| 5.5 | 609.6 | 24.0 |
| 9.5 | 611.3 | 22.0 |
| 13.5 | 609.7 | 24.8 |
| 17.5 | 611.2 | 21.3 |

**Table S8**: Sediment depth indication, chloride and sulfate concentrations of pore water analyses of GeoB19242-6.

| Depth / cmbsf | Chloride / mmol/L | Sulfate / mmol/L |
| --- | --- | --- |
| 1 | 573.5 | 26.1 |
| 5 | 542.4 | 22.7 |
| 9 | 506.1 | 21.7 |
| 13 | 416.8 | 18.2 |
| 17 | 314.3 | 9.4 |

**Table S9**: Sediment depth indication, chloride and sulfate concentrations of pore water analyses of GeoB19242-10.

| Depth / cmbsf | Chloride / mmol/L | Sulfate / mmol/L |
| --- | --- | --- |
| 1.5 | 587.9 | 27.1 |
| 5.5 | 546.5 | 22.6 |
| 9.5 | 521.0 | 22.6 |
| 13.5 | 471.3 | 18.3 |
| 17.5 | 419.8 | 14.1 |

**Table S10**: Sediment depth indication, chloride and sulfate concentrations of pore water analyses of GeoB19242-11.

| Depth / cmbsf | Chloride / mmol/L | Sulfate / mmol/L |
| --- | --- | --- |
| 2 | 525.3 | 23.5 |
| 6 | 414.7 | 14.9 |
| 10 | 292.8 | 7.0 |
| 14 | 185.7 | 1.4 |
| 18 | 145.9 | 0.3 |

**Table S11**: Sediment depth indication, chloride and sulfate concentrations of pore water analyses of GeoB19242-14.

| Depth / cmbsf | Chloride / mmol/L | Sulfate / mmol/L |
| --- | --- | --- |
| 1.5 | 539.4 | 25.8 |
| 5.5 | 438.3 | 17.5 |
| 13.5 | 171.5 | 0.9 |
| 17.5 | 169.2 | 1.1 |

**Table S12**: Sediment depth indication, chloride and sulfate concentrations of pore water analyses of GeoB19242-17.

| Depth / cmbsf | Chloride / mmol/L | Sulfate / mmol/L |
| --- | --- | --- |
| 2 | 243.3 | 4.5 |
| 10 | 160.2 | 1.0 |
| 14 | 128.2 | 0.3 |
| 18 | 160.9 | 0.7 |

**Table S13**: Sediment depth indication, chloride and sulfate concentrations of pore water analyses of GeoB19245-1.

| Depth / cmbsf | Chloride / mmol/L | Sulfate / mmol/L |
| --- | --- | --- |
| 22 | 274.8 | 7.7 |
| 70 | 130.5 | 0.4 |
| 89 | 137.6 | 0.6 |
| 107 | 130.0 | 0.3 |
| 127 | 133.7 | 0.4 |
| 147 | 132.7 | 0.3 |
| 197 | 127.9 | 0.1 |
| 260 | 149.8 | 1.0 |
| 325 | 138.5 | 0.5 |
| 395 | 125.7 | 0.0 |
| 447 | 128.8 | 0.0 |
| 498 | 125.4 | 0.0 |

## References
